# Active *Mycobacterium tuberculosis* infection at a comprehensive cancer center, 2006–2014

**DOI:** 10.1186/s12879-019-4586-y

**Published:** 2019-11-06

**Authors:** Joumana Kmeid, Prathit A. Kulkarni, Marjorie V. Batista, Firas El Chaer, Amrita Prayag, Ella J. Ariza-Heredia, Victor E. Mulanovich, Roy F. Chemaly

**Affiliations:** 1 Department of Infectious Diseases, Infection Control, and Employee Health, The University of Texas MD Anderson Cancer Center, Houston, TX USA; 2 Infectious Diseases Section, Department of Medicine, Baylor College of Medicine, Houston, TX USA; 3 Medical Care Line, Michael E. DeBakey Veterans Affairs Medical Center, Houston, TX USA; 4 University of Maryland Greenebaum Comprehensive Cancer Center Baltimore, Baltimore, MD USA

**Keywords:** Tuberculosis, Stem cell transplantation, Leukemia, Cancer, Pulmonary, Death

## Abstract

**Background:**

Morbidity and mortality from *Mycobacterium tuberculosis* (Mtb) infection remain significant in cancer patients. We evaluated clinical characteristics, management, and outcomes in patients with active Mtb infection at our institution who had cancer or suspicion of cancer.

**Methods:**

We retrospectively examined medical records of all patients with laboratory-confirmed active Mtb infection diagnosed between 2006 and 2014.

**Results:**

A total of 52 patients with laboratory-confirmed active Mtb infection were identified during the study period, resulting in an average rate of 6 new cases per year. Thirty-two (62%) patients had underlying cancer, while 20 (38%) patients did not have cancer but were referred to the institution because of suspicion of underlying malignancy. Among patients with cancer, 18 (56%) had solid tumors; 8 (25%) had active hematologic malignancies; and 6 (19%) had undergone hematopoietic-cell transplantation (HCT). Patients with and without cancer were overall similar with the exception of median age (61 years in cancer patients compared to 53 years in noncancer patients). Pulmonary disease was identified in 32 (62%) patients, extrapulmonary disease in 10 (19%) patients, and disseminated disease in 10 (19%) patients. Chemotherapy was delayed in 53% of patients who were to receive such treatment. Eleven patients (all of whom had cancer) died; 3 of these deaths were attributable to Mtb infection.

**Conclusions:**

Although not common, tuberculosis remains an important infection in patients with cancer. Approximately one-third of patients were referred to our institution for suspicion of cancer but were ultimately diagnosed with active Mtb infection rather than malignancy.

## Background

The worldwide incidence rate of *Mycobacterium tuberculosis* (Mtb) infection has been decreasing for greater than 10 years [1]. However, active Mtb infection remains a serious global infectious disease, with greater than 10 million cases estimated to occur each year [1]. Previous reports have described features of this infection in cancer patients [2–4]. It is generally thought that immunocompromised patients have higher risk of progression from latent Mtb infection to active disease [5, 6]. In a large meta-analysis [7], the rate of developing active Mtb disease was 9-fold higher in patients with hematological malignancies, head and neck cancer, and lung cancer as compared to patients without cancer. Another review of patients with active Mtb infection diagnosed at a comprehensive cancer center suggested that country of origin and type of malignancy were associated with active tuberculosis; foreign-born patients with hematological malignancy had a higher rate of active tuberculosis when compared to U.S.-born patients with solid tumors [8]. In addition, treatment of active Mtb infection can also be challenging in cancer patients because of the potential for drug-drug interactions with antituberculous medications, particularly rifamycins [9].

As a dedicated cancer center, many patients who seek care at our institution come from countries with a high burden of tuberculosis. Two previous studies performed at our institution characterized active Mtb infections over a span of 10 years (1990–2000) and 4 years (2001–2005), respectively [3, 4]. In the first study, De La Rosa et al. reported that the frequency of active Mtb infection was 0.2/1000 new cancer diagnoses and that 63% of patients with active Mtb infection had underlying hematologic malignancy [3]. Interestingly, 4 patients in the study who had been on high-dose corticosteroids died. Clinical isolates available for testing were found to be universally susceptible to all first-line antituberculous drugs. In the second study, Aisenberg GM et al reported the same frequency of active Mtb infection also at 0.2/1000 new cancer diagnoses [4]. Eight (31%) out of the 26 patients with active Mtb infection in the study had been referred to our institution for evaluation of a suspicious mass thought to be malignant but were subsequently diagnosed with active Mtb infection.

Because of changes in cancer incidence, management, and survival over recent years, the scope of active Mtb infection in cancer patients specifically might also have changed. Therefore, the purpose of the current study was to re-evaluate the characteristics and outcomes of a recent cohort of patients with active Mtb infection receiving care at our institution over a 9-year period of time (2006–2014).

## Methods

### Patient identification

In this retrospective study, we searched the institution’s Infection Control database to identify patients with laboratory-confirmed active Mtb infection diagnosed between January 2006 and December 2014. Data collected from medical records of these patients included demographic information such as age, gender, race, and country of origin. Medical information obtained included presence and type of underlying malignancy, presence of underlying autoimmune or rheumatologic disease, receipt of hematopoeitic-cell transplantation (HCT), use of systemic corticosteroids within 30 days prior to Mtb diagnosis, use of any chemotherapeutic or immunosuppressive agents within 6 months prior to Mtb diagnosis, results of laboratory and radiographic studies, and previous testing and/or treatment for latent or active Mtb infection. Data on treatment and outcomes, including clinical and radiographic improvement after treatment with antituberculous drugs, side effects from medications, delay in chemotherapy, and death, were also collected. Information about the overall number of new cancer diagnoses at our center was obtained from the institutional data registry.

### Definitions, organism identification, and susceptibility testing

A case of active Mtb infection was defined as any patient with clinical signs and symptoms and/or radiographic features compatible with active Mtb infection and isolation of Mtb by culture from any sputum, body fluid, and/or tissue specimen. We excluded patients with possible active Mtb disease based on clinical and radiographic data but without concomitant microbiological evidence of infection. In addition, we included 2 patients who had tissue specimens that tested positive for Mtb by 16S ribosomal DNA sequencing. The sequencing procedure was developed and its performance characteristics verified by our institution’s microbiology laboratory, a Clinical Laboratory Improvement Amendments (CLIA)-certified and College of American Pathologists-accredited clinical diagnostic laboratory.

Drug susceptibility testing was performed at a reference laboratory using the BACTEC radiometric system (Becton, Dickinson, and Company, Towson, MD). Neutropenia was defined as an absolute neutrophil count lower than 500/μL at time of collection of the positive mycobacterial culture.

A list of countries deemed to have a high burden of tuberculosis was obtained from the 2017 Global Tuberculosis Report, published by the World Health Organization (WHO) [1]. History of Mtb exposure was categorized into 3 groups: 1) positive history of Mtb exposure, defined as close contact with a person with documented active pulmonary Mtb infection; 2) probable history of Mtb exposure, defined as high risk for possible Mtb exposure because of occupation in a healthcare setting, HIV infection, and/or prior injection drug use, incarceration, or homelessness; and 3) negative history of Mtb exposure, defined as any individual that did not fit into the first 2 groups. Mortality was attributed to Mtb if persistent or progressive Mtb infection with respiratory failure was present at the time of death. Disseminated disease was defined as evidence of infection involving two or more noncontiguous body sites.

### Statistical analysis

Data are presented as median values (± range) and absolute count (percentage), as appropriate. Differences in categorical variables between patients with or without cancer were evaluated with univariate analysis using Fisher’s exact test. A two-sided *P* value <.05 was considered statistically significant. Data were analyzed using IBM SPSS statistical software 24.

## Results

### Clinical characteristics

**Fig. 1:**
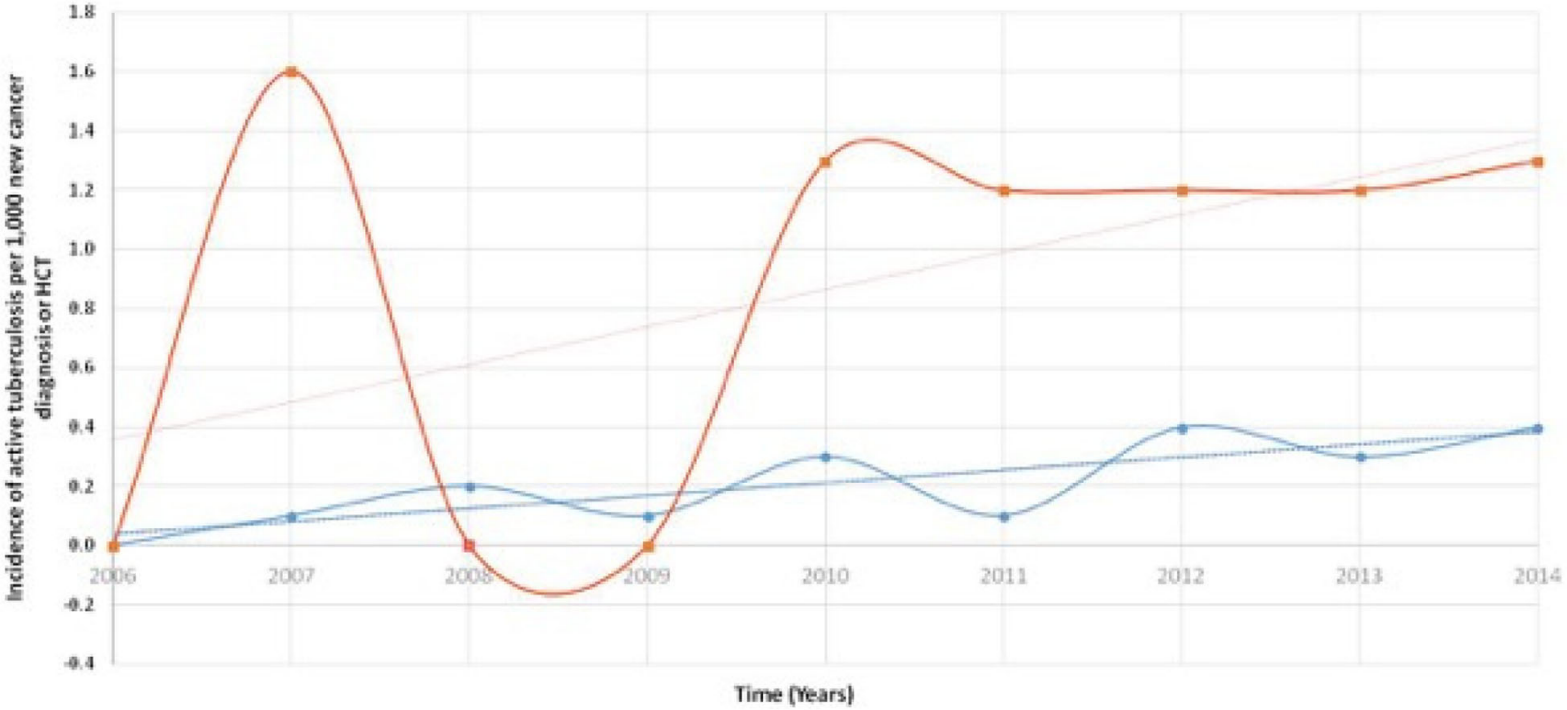
Annual incidence of active tuberculosis infection in cancer patients from 2006 to 2014 at University of Texas MD Anderson Cancer Center. The red and blue dotted line represents the trend in incidence rate, in HCT recipients and in cancer patients respectively, over the same period of time

We identified 52 patients with active Mtb infection diagnosed between January 2006 and December 2014, resulting in an average of 6 new cases per year and an overall incidence rate of 0.2 cases per 1000 new cancer diagnoses compared to 1.7 cases per 1000 new HCT (Fig. 1). Table 1 compares the characteristics and outcomes of active Mtb infection in patients with or without a cancer diagnosis. The two groups were similar except that patients with cancer were significantly older with a median age of 61 years (range: 33–85) as compared to patients without cancer (median age of 53 years [range: 16–78]).

**Table 1:** Characteristics and outcomes of patients with active tuberculosis infection with or without cancer diagnosis at University of Texas MD Anderson Cancer Center from 2006 to 2014

Characteristic	Without cancer diagnosis *n* = 20 (%)	With cancer diagnosis *n* = 32 (%)	*p* value
Age	53 (16–78)	61 (33–85)	0.016
Gender			
Male	11 (55)	25 (78)	0.067
Female	9 (45)	7 (22)	
Race			0.213
White	4 (20)	9 (28)	
Black	0	5 (16)	
Hispanic	5 (25)	5 (16)	
Asian	11 (55)	13 (40)	
Underlying conditions			
Solid Tumor	_	18 (56)	
Leukemia	_	6 (19)	
Lymphoma	_	2 (6)	
Hematopoetic cell transplantation	_	6 (19)	
Suspicion for cancer	17 (85%)	_	
Human Immunodeficiency Virus	1 (5%)	_	
Psoriasis	1 (5%)	_	
Healthcare worker	1 (5%)	_	
Cancer Status			
Active disease	_	16 (50)	
Complete remission	_	15 (47)	
Partial remission	_	1 (3)	
Corticosteroid use	3 (15)	7 (22)	0.541
Chemotherapy/Immunosuppressants	1 (5)^a^	19 (59)	
Endemic region for Tuberculosis	14 (70)	15 (47)	0.15
History of exposure to Tuberculosis			0.47
Yes	8 (40)	10 (31)	
Probable	3 (15)	3 (9)	
Previous treatment of latent Tuberculosis			
	3^d^	3^e^	
Site of infection			0.84
Pulmonary	12 (60)	20 (60)	
Extrapulmonary	5 (25)	5 (16)	
Lymph nodes	3 (15)	4 (13)	
Peritoneum	1 (5)	_	
Bones and joints	1 (5)	_	
Other^b^	_	1 (5)	
Disseminated	3 (15)	7 (22)	
Radiologic findings^c^			
Cavitary	5 (25)	6 (19)	0.591
Nodular	9 (45)	15 (47)	0.895
Death	0	11 (34)	0.0036
Attributed to Tuberculosis	0	3 (27)	0.276

^a^Immunomodulators for psoriasis

^b^Pelvic mass

^c^Chest imaging available for 24 patients

^d^2 complete, 1 incomplete

^e^1 complete, 2 incomplete

Overall, 36 patients (69%) were male (11 [55%] without cancer and 25 [78%] with cancer). A plurality of patients were Asian (24 [46%]), followed by non-Hispanic white (13 [25%]), Hispanic (10 [(19%]), and black (5 [10%]). A total of 31 patients (60%) were foreign-born, with 29 (56%) originating from countries with a high burden of tuberculosis [14 (70%) without cancer and 15 (47%) with cancer], including the Philippines (9), India (5), China (4), Vietnam (2), Pakistan (2), and Thailand (1). Eighteen patients (35%) had a positive history of Mtb exposure, and 6 patients (11%) had probable exposure.

Additionally, 6 patients (12%) had previously been treated for latent Mtb infection; 3 patients completed an approved regimen, while the other 3 patients’ treatment courses were interrupted. Among the patients who had previously received complete courses of treatment for latent Mtb infection, one patient had acute myeloid leukemia and was receiving ruxolitinib at the time of diagnosis; one patient had been referred for suspicion of malignancy and was not receiving any immunosuppressive medications; and one patient had psoriasis for which she received infliximab and methotrexate 3 months prior to diagnosis. Among the patients who had previously received incomplete course of therapy for latent Mtb infection, one patient had active breast cancer and Crohn’s disease and was receiving 6-mercaptopurine at the time of diagnosis; one patient had undergone matched unrelated donor HCT 2 years prior for multiple myeloma and was receiving tacrolimus; and one patient who had been referred for suspicion of malignancy was not receiving any immunosuppressive therapy. One additional patient had recurrent active Mtb infection after he had completed an appropriate course of therapy for pulmonary disease approximately 6 months prior. This patient had underlying multiple sclerosis and was on no immunosuppressive therapy at the time of presentation; he had been referred to our institution because of suspicion of malignancy.

A total of 32 patients (62%) had underlying malignancy, 18 (56%) with different types of solid tumors, including anaplastic oligodendroglioma, breast cancer, esophageal cancer, basal cell carcinoma of the cheek and nose, neuroendocrine carcinoma, prostate cancer, renal cell carcinoma, pancreatic cancer, rectal carcinoma, lung cancer, laryngeal cancer, colon cancer, thyroid carcinoma and urothelial carcinoma; 8 (25%) with hematologic malignancies (6 patients with leukemia and 2 with lymphoma); and 6 (19%) who had undergone HCT (2 autologous and 4 allogeneic). Sixteen patients with cancer (31%) were in remission at the time of Mtb diagnosis. Seven of 32 patients (22%) had received corticosteroids in the month prior to diagnosis, and 19 out of 32 (59%) had received chemotherapy or other immunomodulating agents in the 6 months prior to diagnosis.

Among the 20 patients (38%) without cancer, the vast majority (17 [85%]) were suspected of having a malignancy but were ultimately diagnosed with active Mtb infection. Three of the patients suspected to have malignancy had received corticosteroids within the month prior to presentation for different indications; 1 patient had multiple sclerosis but was not receiving any immunosuppressive therapy at the time of presentation; and 1 patient had psoriasis and had received infliximab and methotrexate approximately 3 months prior. Of the 3 remaining patients without cancer who were not suspected of having a malignancy, one had underlying HIV infection and presented with pancytopenia; one had psoriasis; and one was a healthy healthcare worker.

### Clinical presentation

The clinical presentation of Mtb infection varied amongst our patients, as shown in Table 1. The majority of patients had pulmonary disease (12 [60%] among non-cancer patients and 20 [63%] among cancer patients); 10 had extrapulmonary disease (5 [25%] among non-cancer patients and 5 [16%] among cancer patients); and 10 patients had disseminated disease (3 [15%] among non-cancer patients and 7 [22%] among cancer patients). Of the 10 patients with disseminated disease, 9 had combined pulmonary and extrapulmonary infection, and 1 had extrapulmonary infection in 2 noncontiguous body sites (abdomen and prostate). The clinical presentation of patients with extrapulmonary Mtb infection included a pelvic mass, osteomyelitis or vertebral lesions, peritoneal disease, and lymphadenitis. With regard to radiographic appearance of disease, chest imaging (most frequently a computed tomographic [CT] scan) showed a variety of findings, including nodules (24 cases), cavitary lesions (11 cases), mass (3 cases), non-nodular infiltrates (7 cases), and pleural disease (5 cases).

### Laboratory testing

Table 2 shows the differences in diagnostic testing between non-cancer and cancer patients. Overall, a smear for acid-fast bacilli (AFB) was positive in 7/51 patients (4/20 [25%] without cancer and 3/31 [10%] with cancer). Types of samples included 3 sputum specimens, 2 lung tissue specimens, 1 bronchoalveolar lavage (BAL) fluid specimen, and 1 fine needle aspirate (FNA) from a lymph node. Among the 3 tissue specimens on which molecular testing was performed, 2 samples were positive and 1 negative. Mycobacterial cultures were done on tissue specimens in 19 cases (37%), BAL fluid in 14 cases (27%), other body fluid or FNA in 10 cases (19%), and sputum in 7 cases (13%); 2 positive cultures were obtained simultaneously from BAL fluid and FNA in the same patient.

**Table 2:** Comparison of different diagnostic tests for patients with active tuberculosis infection at University of Texas MD Anderson Cancer Center from 2006 to 2014

Patients	Without cancer diagnosis *N* = 20 (%)	With cancer diagnosis *N* = 32 (%)
Positive AFB smear	5 (25)	6 (19)
Positive TB Culture	20 (100)	32 (100)
Sputum	1 (5)	6 (19)
BAL	6 (30)	9 (28)
FNA	2 (10)	9 (28)
Tissue	11 (55)	8 (25)
Positive TB PCR^a^	1	1
Positive IGRA	7/8^b^ (88)	9/16^c^ (56)
T-SPOT.*TB*	0	1
QFT.*TB*	7	8
Negative IGRA	1/8^b^ (13)	3/16^c^ (19)
T-SPOT.*TB*	1	0
QTF.*TB*	0	3
Inconclusive IGRA	0	4/16^c^ (25)
T-SPOT.*TB*	0	0
QTF.*TB*	0	4

^*a*^ *3 TB PCR were performed*

^*b*^ *8 in non-cancer patients*

^*c*^ *16 cancer patients*

*Abbreviations: AFB, Acid fast bacilli; TB, tuberculosis; BAL, bronchoalveolar lavage; FNA, fine needle aspirate; PCR, polymerase chain reaction; IGRA, interferon gamma release assay; QFT, QuantiFERON*

Twenty-four patients were tested with an interferon-gamma release assay (IGRA) at the time of diagnosis, 22 with Quantiferon TB-Gold® (QFT.*TB*, Qiagen) and 2 with T-Spot.TB® (T-spot. *TB*, Oxford Immunotec, Inc., Memphis, TN). Sixteen IGRA tests (67%) showed positive results; 4 (17%) were negative; and 4 (17%) were inconclusive (either indeterminate or invalid).

### Antimicrobial susceptibility

Drug susceptibility testing was performed on all clinical isolates. The majority of isolates, 73%, were susceptible to all first-line antituberculous agents, while 14 isolates (27%) were resistant to at least one agent (3 [15%] from patients without cancer and 11 [34%] from patients with cancer). Seven of these 14 isolates (50%) were resistant to pyrazinamide, 3 (22%) to streptomycin, 2 (14%) to isoniazid, and 2 (14%) to both isoniazid and streptomycin. No multidrug resistant (MDR) Mtb isolates, defined as resistance at least to isoniazid and rifampin, were identified in this cohort.

### Management and outcomes

A total of 49 patients received therapy for active Mtb infection. The remaining 3 patients were non-cancer patients who presented with a lung mass (2 patients) or pancytopenia (1 patient); these patients ultimately received medical care elsewhere. There was no difference between non-cancer and cancer patients with regard to treatment characteristics. The median time from obtaining a positive mycobacterial culture to start of therapy was 21 days (range: 5–72 days). The median treatment duration of the 32 patients with complete data available was 6 months.

Ten of 43 patients (23%) for whom data were available developed significant side effects from therapy; 9 of these 10 had cancer. However, all patients were able to restart their medications or change regimens in order to complete a full treatment course. Overall, twenty-eight of 33 patients (85%) for whom complete data were available completed their therapy. Among the 5 patients who were not able to complete a full course of therapy, 4 had treatment interruption because of death, and one patient received only 4 months of therapy for extrapulmonary tuberculosis because of concern about drug-drug interactions between chemotherapy and antituberculous medications. Chemotherapy was delayed in 9 out of 17 patients (53%) who required such treatment. Forty out of 42 patients (95%) for whom data were available had documented initial clinical and/or radiographic improvement with treatment.

Overall mortality in this cohort was 21%, with 11 deaths out of 52 patients; all 11 deaths were in patients with cancer. A total of 4 deaths occurred while on antituberculous therapy; 2 of these 4 patients died within 12 days of initiation of therapy. Death was attributed to active Mtb infection in 3 patients (6%). Two patients had advanced solid tumors (esophageal cancer and neuroendocrine tumor) and were receiving palliative care; they died with disseminated Mtb infection. The third patient had relapsed multiple myeloma after autologous HCT 6 years prior to diagnosis of pulmonary tuberculosis. Of note, 2 of the 3 patients with death attributable to active Mtb infection had isolates that were resistant to at least 1 first-line agent (1 to pyrazinamide and 1 to both isoniazid and streptomycin).

## Discussion

In this retrospective study, we report the characteristics and outcomes of patients diagnosed with active Mtb infection at a comprehensive cancer center over a 9-year period of time. The frequency of active Mtb infection was found to be 0.2 per 1000 new cancer diagnoses, comparable to the previous incidence rates determined in 1990–2000 and in 2001–2005 at our institution [3, 4]. However, there was a trend noted in recent years of a higher annual incidence of Mtb infections in patients cared for at our institution, which which can likely be explained by the higher number of patients referred from countries where latent or active Mtb infections are more prevalent. Interestingly, the incidence of active Mtb infections in HCT recipients was higher than in other cancer patients. A study from Taiwan, a country with a high burden of TB, showed an incidence of 6.8 per 1000 person-years in HCT recipients [10], in contrast to a study from a US institution where no cases of tuberculosis were reported in a cohort of 2531 HCT recipients from 2010 to 2015 [11]. Table 3 shows the comparison of findings from the current study and the 2 prior studies conducted at our institution.

**Table 3:** Comparison of findings in patients with cancer and active tuberculosis infection at University of Texas MD Anderson Cancer Center from 1990 to 2014

Period of study	1990–2000 [3] *N* = 30 (%)	2001–2005 [4] *N* = 18 (%)	2006–2014^#^ *N* = 32 (%)
Incidence (per 1000 new cancer diagnosis)	0.2	0.2	0.2
Treated for tuberculosis	29 (97)	14 (78)	19^b^ (79)
Radiographic improvement	Not available	8^a^ (80)	29^c^ (94)
Responded to therapy	23 (79)	18 (100)	29^c^ (94)
Death attributed to tuberculosis	7 (23)	2 (11)	3 (9)

^#^ Current study

^a^ data available for 10 patients

^b^ data available for 24 patients

^c^ data available for 31 patients

An important finding in the present study was that nearly 40% of patients with active Mtb infection were referred to our institution because of initial suspicion for malignancy but were ultimately diagnosed with tuberculosis. This finding corresponds to the most recently conducted study at our institution on this subject, in which 31% of patients with active Mtb infection had initially been referred because of suspicion of cancer [4]. It therefore remains essential to pursue tissue diagnosis in instances when tuberculosis is a consideration because of its wide spectrum of presentation and radiographic overlap with malignant processes.

We found that 60% of the study population was comprised of patients who were born in countries outside of the United States; 56% of patients were from countries with a high burden of tuberculosis. This result is comparable to findings from the two prior studies conducted at our institution, in which the percentages of foreign-born patients with active Mtb infection were 60 and 38% [3, 4]. This finding is also similar to the general U.S. population; according to data from the Centers for Disease Control and Prevention, in 2017, approximately 70% of new tuberculosis cases in the United States occurred in foreign-born persons, and the incidence rate of tuberculosis among foreign-born persons was 15 times the incidence rate among U.S.-born persons [12]. As a referral comprehensive cancer center that provides care for a large number of international patients, considering tuberculosis in the appropriate clinical context is important because of the higher prevalence amongst foreign-born persons.

Similar to previous studies conducted at our institution, the clinical presentation of active Mtb infection was found to be diverse with a mix of pulmonary, extrapulmonary, and combined pulmonary/extrapulmonary disease [3, 4]. Extrapulmonary disease involved many different anatomical sites, including peritoneum, bone, pelvic organs, and lymph nodes. Radiographic findings were also varied, with active Mtb infection appearing on chest imaging as nodules, cavitation, masses, and other non-cavitary lung infiltrates. Importantly, in WHO’s report on the global burden of tuberculosis, incident extrapulmonary cases of active Mtb infection represented 15% of the total reported number [1]. By contrast, in the present study, 21% of active Mtb cases were extrapulmonary, and an additional 17% of patients had combined pulmonary and extrapulmonary disease.

Regarding use of IGRA as an adjunct test to help diagnose active Mtb infection, QFT.*TB* was the predominant type of IGRA used at that time in our institution. We found that 16% of IGRAs were negative despite ongoing active infection; 3 negative tests occurred in cancer patients and 1 in a non-cancer patient. Additionally, 4/24 (17%) of IGRA tests were indeterminate; all 4 tests occurred in cancer patients. Although there are no studies to our knowledge that have specifically evaluated the utility of IGRA testing for diagnosis of active Mtb infection in cancer patients, two prior studies have compared an ELISPOT assay (T-spot.*TB*, Oxford Immunotec, Inc., Memphis, TN) with tuberculin skin test (TST) for diagnosis of latent tuberculosis infection (LTBI) in immunocompromised patients [13, 14]. Piana et al found that 61/138 (44%) patients with hematological malignancy who had been exposed to a case of smear-positive active pulmonary Mtb infection had a positive T-spot.*TB*, while only 24 (17%) had a positive TST [13]. In another study, among 62 patients with hematologic malignancy and a positive T-spot.*TB*, 41 received TST, among which only 20 (49%) were positive [14]. An additional study from 2009 that compared TST with both T-spot.*TB* and QFT.*TB* found that amongst 95 patients with hematologic malignancy, 10/95 (11%) had a positive TST; 25/95 (26%) had a positive T-spot; and 17/95 (18%) had a positive QFT [15]. Although IGRA testing is not licensed for diagnosis of active TB, our results suggest that IGRA tests remain an important but not wholly reliable test to diagnose active Mtb infection in cancer patients; this is consistent with prior findings on this subject in general, not necessarily in cancer patients [16]. Importantly, a positive IGRA test cannot differentiate between LTBI and active TB.

Most patients (28 out of 33 for whom data were available) were able to complete a full course of antituberculous therapy; the median treatment duration was 6 months (Table 1). In the 10 patients who experienced side effects from antituberculous therapy prominent enough to require treatment interruption, all patients were ultimately able to complete a full course of treatment. In addition, only 1 patient had to specifically stop therapy for active Mtb infection because of drug-drug interactions related to chemotherapy. These results indicate that for the vast majority of patients at a tertiary-care cancer center, treatment of active Mtb disease is readily possible, despite concerns about medication toxicities and drug-drug interactions.

Our study had certain limitations. First, it was a retrospective study using only information obtained from medical records. Second, certain data, mainly related to treatment duration and outcomes, were unavailable. This was particularly a problem among noncancer patients, many of whom received the majority of their care and antituberculous treatment at other institutions after diagnosis. This limits conclusions that might be drawn regarding outcomes between cancer and non-cancer patients. Third, the sample in this study was limited to one particular tertiary-care specialty institution and therefore might not be generalizable to other areas around the country or other types of institutions.

## Conclusions

Active Mtb infection remains an important diagnostic consideration at our institution. Many patients without a known cancer diagnosis with active Mtb infection were initially thought to have cancer. It is important for clinicians to consider tuberculosis when the presenting clinical syndrome and epidemiological exposure are compatible with this diagnosis, including patients referred for suspicion of cancer. Prompt diagnosis of active Mtb infection may have important infection control implications, particularly at a tertiary cancer center with many vulnerable immunosuppressed patients.

## Data Availability

All data and materials described in this manuscript are available for other researchers (Please contact Dr. Roy F. Chemaly).

## Abbreviations

AFB: Acid-fast bacilli
BAL: Bronchoalveolar lavage
CLIA: Clinical Laboratory Improvement Amendments
CT: Computed tomographic
FNA: Fine needle aspirate
HCT: Hematopoietic-cell transplantation
HIV: Human immunodeficiency virus
IGRA: Interferon-gamma release assay
LTBI: Latent tuberculosis infection
MDR: Multidrug resistant
Mtb: *Mycobacterium tuberculosis*
WHO: World Health Organization
